# Exploring the clinical benefit of ventilation therapy across various patient groups with COVID-19 using real-world data

**DOI:** 10.1038/s41598-023-37912-5

**Published:** 2023-07-03

**Authors:** Mohsen Abbasi-Kangevari, Ali Ghanbari, Mohammad-Reza Malekpour, Seyyed-Hadi Ghamari, Sina Azadnajafabad, Sahar Saeedi Moghaddam, Mohammad Keykhaei, Rosa Haghshenas, Ali Golestani, Mohammad-Mahdi Rashidi, Nazila Rezaei, Erfan Ghasemi, Negar Rezaei, Hamid Reza Jamshidi, Bagher Larijani

**Affiliations:** 1 Non-Communicable Diseases Research Center, Endocrinology and Metabolism Population Sciences Institute, Tehran University of Medical Sciences, Tehran, Iran; 2 Kiel Institute for the World Economy, Kiel, Germany; 3 Division of Cardiology, Department of Medicine, The Johns Hopkins University School of Medicine, Baltimore, MD USA; 4 School of Public Health, University of Alberta, Edmonton, AB Canada; 5 Endocrinology and Metabolism Research Center, Endocrinology and Metabolism Clinical Sciences Institute, Tehran University of Medical Sciences, Tehran, Iran; 6 Department of Pharmacology, Faculty of Medicine, Shahid Beheshti University of Medical Sciences, Tehran, Iran

**Keywords:** Health services, Medical ethics, Public health, Epidemiology, Outcomes research, SARS-CoV-2

## Abstract

Scarcity of ventilators during COVID-19 pandemic has urged public health authorities to develop prioritization recommendations and guidelines with the real-time decision-making process based on the resources and contexts. Nevertheless, patients with COVID-19 who will benefit the most from ventilation therapy have not been well-defined yet. Thus, the objective of this study was to investigate the benefit of ventilation therapy among various patient groups with COVID-19 admitted to hospitals, based on the real-world data of hospitalized adult patients. Data used in the longitudinal study included 599,340 records of hospitalized patients who were admitted from February 2020 to June 2021. All participants were categorized based on sex, age, city of residence, the hospitals' affiliated university, and their date of hospitalization. Age groups were defined as 18–39, 40–64, and more than 65-year-old participants. Two models were used in this study: in the first model, participants were assessed by their probability of receiving ventilation therapy during hospitalization based on demographic and clinical factors using mixed-effects logistic regression. In the second model, the clinical benefit of receiving ventilation therapy among various patient groups was quantified while considering the probability of receiving ventilation therapy during hospital admission, as estimated in the first model. The interaction coefficient in the second model indicated the difference in the slope of the logit probability of recovery for a one-unit increase in the probability of receiving ventilation therapy between the patients who received ventilation compared to those who did not while considering other factors constant. The interaction coefficient was used as an indicator to quantify the benefit of ventilation reception and possibly be used as a criterion for comparison among various patient groups. Among participants, 60,113 (10.0%) cases received ventilation therapy, 85,158 (14.2%) passed away due to COVID-19, and 514,182 (85.8%) recovered. The mean (SD) age was 58.5 (18.3) [range = 18–114, being 58.3 (18.2) among women, and 58.6 (18.4) among men]. Among all groups with sufficient data for analysis, patients aged 40–64 years who had chronic respiratory diseases (CRD) and malignancy benefitted the most from ventilation therapy; followed by patients aged 65 + years who had malignancy, cardiovascular diseases (CVD), and diabetes (DM); and patients aged 18–39 years who had malignancy. Patients aged 65 + who had CRD and CVD gained the least benefit from ventilation therapy. Among patients with DM, patients aged 65 + years benefited from ventilation therapy, followed by 40–64 years. Among patients with CVD, patients aged 18–39 years benefited the most from ventilation therapy, followed by patients aged 40–64 years and 65 + years. Among patients with DM and CVD, patients aged 40–64 years benefited from ventilation therapy, followed by 65 + years. Among patients with no history of CRD, malignancy, CVD, or DM, patients aged 18–39 years benefited the most from ventilation therapy, followed by patients aged 40–64 years and 65 + years. This study promotes a new aspect of treating patients for ventilators as a scarce medical resource, considering whether ventilation therapy would improve the patient's clinical outcome. Should the prioritization guidelines for ventilators allocation take no notice of the real-world data, patients might end up being deprived of ventilation therapy, who could benefit the most from it. It could be suggested that rather than focusing on the scarcity of ventilators, guidelines focus on evidence-based decision-making algorithms to also take the usefulness of the intervention into account, whose beneficial effect is dependent on the selection of the right time in the right patient.

## Introduction

The coronavirus disease-2019 (COVID-19) pandemic has far officially claimed more than 5.22 million lives worldwide^1^. Acute respiratory distress syndrome (ARDS), a common complication of COVID-19 among critically ill patients, requires medical management involving ventilation therapy. Of all patients diagnosed with COVID-19, 17% to 35% would be hospitalized at intensive care units (ICUs)^2,3^, and 9% to 19% would require invasive mechanical ventilation^2,4^. The availability of ICU beds varies widely between countries, even among the wealthiest countries^5^.

While COVID-19 continues to place extraordinary demands on healthcare systems, resulting in severe shortages of essential resources and services^6^, the scarcity of ventilators could be the most challenging, as there is typically limited time if mechanical ventilation is vital^7^. The estimated number of available invasive mechanical ventilators in various countries would not be adequate to serve all clinically eligible patients during the pandemic^8^.

Research has been ongoing to investigate the main principles for allocating scarce medical resources during pandemics^9–11^. Medical experts working at the COVID-19 care units interact with patients of different socioeconomic, clinical, paraclinical, and overall health statuses. While physicians should not be faced with situations where they would be obliged to decide which patient to treat due to the risk of human error as well as the double-burden of life-long emotional toll, the pandemic has increased the likelihood of such dilemmas, especially in settings with limited resources^12^. Thus, prioritization recommendations and guidelines are under development in the hope of helping physicians, especially those less experienced, with the real-time decision-making process based on the resources and contexts^6,13^. Serious discussions on the ethical considerations of ventilator allocation were also raised during the pandemic. Utility (maximizing benefits) and equity (distributive justice) were two concerns raised in decision making^14,15^ in such dilemma which has also been considered to be “the toughest triage”^7^. From a utilitarian perspective, saving the most lives or saving the most life-years by allocation of ventilation to those with higher survival could guide rationing^7,14,15^.

Nevertheless, there is not much information about ventilation therapy for patients with COVID-19. Drawing from previous World Health Organization (WHO) guidelines, there are recommendations to indicate which patients with hypoxemic respiratory failure should be considered for non-invasive ventilation and prioritize in settings with limited resources^16^. It remains challenging yet imperative to prioritize therapy to patients who will benefit the most from it considering availability and risk, considering the increased risk of infection transmission when the patient undergoes endotracheal intubation and non-invasive ventilation^17^. Determining which patients with COVID-19 would benefit the most from ventilation therapy could help optimize the current ventilator allocation guidelines. Thus, the objective of this study was to investigate the benefit of ventilation therapy among various patient groups with COVID-19 admitted to hospitals, based on the real-world data of hospitalized adult patients.

## Material and methods

### Ethics

This work was supported by the WHO EMRO Office (EMRO) (Grant No. 202693061). The study methodology conformed to Helsinki Declaration standards as revised in 1989. The ethics committee of Endocrinology and Metabolism Research Center, Tehran University of Medical Sciences, Tehran, Iran, approved this study under the reference number IR.TUMS.EMRI.REC.1400.034. The data used in the study did not include any identifiable personal information of participants, and the confidentiality of the data and the results are preserved.

### Overview

Data used included 599,340 records of hospitalized patients with COVID-19 in Iran who were admitted from February 2020 to June 2021. Patients were categorized based on sex, age, city of residence, the hospitals' affiliated university, date of hospitalization, and comorbidities. First, the probability of patients’ ventilation therapy during hospitalization was calculated. Then, patients’ survival was assessed and the clinical benefit of ventilation therapy among various patient groups was quantified while considering the probability of receiving ventilation therapy during hospital admission, as estimated in the first model.

### Data source and variables

Data of this longitudinal study were retrieved from the Iranian COVID-19 registry provided by the Ministry of Health and Medical Education, which was gathered from hospitals and included patients with COVID-19 in Iran from the early days of the pandemic. Data used in the current study included 599,340 records of hospitalized patients who were admitted from February 2020 to June 2021. The study variables included the patients' age; sex; underlying conditions, including diabetes mellitus (DM), cardiovascular diseases (CVD), chronic respiratory disease (CRD), malignancy; receiving ventilation therapy; and COVID-19 outcomes, including recovery or death.

### Case definitions

DM, CVD, CRD, and malignancy were obtained from patients’ self-reported medical history. The diagnosis of COVID-19 was made by physicians based on a positive Real-Time Reverse Transcription Polymerase Chain Reaction (RT-PCR) result for SARS-CoV-2, or clinical suspicion defined as (1) at least two of the following symptoms lasting for at least 48 h: fever (axillary temperature ≥ 37.5 °C), chills, sore throat, stuffy nose, myalgia, fatigue, headache, nausea or vomiting, or diarrhea or (2) at least one respiratory sign or symptom (including cough, shortness of breath), new olfactory or taste disorder, or radiographic evidence of COVID-19–like pneumonia.

### Data analysis

#### Variables

All participants were categorized based on sex, age, city of residence, the hospitals' affiliated university, and their date of hospitalization. Age groups were defined as 18–39, 40–64, and more than 65-year-old participants. The affiliated university were assessed due to the possibility of using disparate approaches and guidelines regarding ventilator allocation policies. The date of hospitalization was also included due to the paramount importance of considering the scarcity of vital equipment at the peak of the COVID-19 epidemic surge. The intervals included in the analysis were as follows: February–March 2020, April–May 2020, June-July 2020, August–September 2020, November–December 2020, January–February 2021, March–April 2021, and May–June 2021. In addition to demographic annotations, patients' data were further assessed for comorbidities and underlying/clinical conditions, which included CRD, CVD, DM, and malignancies.

### Statistical methods

Two models were used in this study: in the first model, participants were assessed by their probability of receiving ventilation therapy during hospitalization based on demographic and clinical factors using mixed-effects logistic regression. In the second model, the clinical benefit of receiving ventilation therapy among various patient groups was quantified while considering the probability of receiving ventilation therapy during hospital admission, as estimated in the first model.

#### Estimating the probability of ventilation therapy

First, we used a mixed-effects logistic regression model^18^ to estimate the probability of receiving ventilation therapy among patients. The response variable was binary, with "one" representing receiving ventilation therapy. The effects of time intervals, age groups and affiliated university were considered as random intercept effects. Sex, ICU admission, CRD, malignancy, CVD, and DM were random intercept effects that varied among different age groups, as presented in the following:

$$\begin{aligned} & \log \left( {\frac{\pi }{1 - \pi }} \right) = intercept + u_{affiliated\;university } + u_{admission\;time} + u_{{{\text{i}} ntercept_{l} }} + u_{{sex_{l} }} + u_{{ICU\;admission_{l} }} + u_{{CRD_{l} }} + u_{{malignancy_{l} }} + u_{{CVD_{l} }} + u_{{DM_{l} }} + \in \\ & \pi = probability\;of\;receiving\;ventilation\;therapy \\ & l = 1, \ldots ,n_{l} ;indicate\;age\;group \\ & u_{affiliated\,university } \sim Normal\left( {0,\sigma_{affiliated\;university} } \right) \\ & u_{time} \sim Normal\left( {0,\sigma_{time} } \right) \\ & \left( {\begin{array}{*{20}l} {u_{{sex_{l} }} } \hfill \\ {u_{{ICU_{l} }} } \hfill \\ {u_{{CRD_{l} }} } \hfill \\ {u_{{malignancy_{l} }} } \hfill \\ {u_{{CVD_{l} }} } \hfill \\ {u_{{DM_{l} }} } \hfill \\ {u_{{intercept_{l} }} } \hfill \\ \end{array} } \right)\sim Multivariate\;Normal\left( {0,{{\varvec{\Sigma}}} } \right) \\ & {\Sigma }is\;a\;variance - covariance\;matrix \\ & \in \sim Normal\left( {0,\sigma } \right) \\ \end{aligned}$$

#### Estimating the probability of recovery

To investigate the extent of benefit among patients with various underlying conditions, the uneven chance of receiving ventilation therapy due to the time of admission, hospital equipment, or resource allocation guidelines used needed to be addressed. First, we divided the patients into 48 groups based on their age groups and underlying conditions, including CRD, malignancy, CVD, DM. Then, considering the high sample size and to simplify the modeling process, a logistic generalized linear model was fitted separately for each group. The response variable was binary with "one" representing recovery. Also, the admission province, admission time, patient sex, and ICU admission were the independent variables. The last term of the model was the interaction between a binary variable, with "one" representing receiving ventilation therapy, and a continuous variable indicating the probability of receiving ventilation therapy obtained from the first model. This interaction gives away two main effects and one interaction coefficient, as presented in the following:

$$\begin{aligned} & {\text{outcome}}\sim {1} + {\text{icu}} + {\text{affiliated}}\;{\text{university}} + {\text{time}} + {\text{sex}} + {\text{ventilation}}\;{\text{therapy}}*{\text{probability}}\;{\text{of}}\;{\text{receiving}}\;{\text{ventilation}}\;{\text{therapy}} \\ & \log \left( {\frac{\pi }{1 - \pi }} \right) = intercept + {\text{icu }} + {\text{affiliated}}\;{\text{university}} + {\text{time}}.{\text{period}} + {\text{sex}} + {\text{ventilator}}.{\text{therapy*probability}}\;{\text{of}}\;{\text{receiving}}\;{\text{ventilation}}\;{\text{therapy}} + \in \\ & \pi = probability\;of\;recovery \\ & \in \sim Normal\left( {0,\sigma } \right) \\ \end{aligned}$$

#### Main effects

The first main effect indicated the ratio of the odds for recovery among patients who received ventilation therapy to the odds for those who did not, while considering other factors constant. The second main effect indicated the ratio of the odds for recovery for a one-unit increase (“zero” probability represents not receiving ventilation therapy, while “one” represents receiving ventilation therapy) in the probability of receiving ventilation therapy, while considering other factors constant.

#### Interaction coefficient

The interaction coefficient indicated the difference in the slope of the logit probability of recovery for a one-unit increase in the probability of receiving ventilation therapy between the patients who received ventilation compared to those who did not while considering other factors constant. We considered the positive and significant coefficient values to represent the benefit of receiving ventilation for patients who receive ventilation compared to those who did not. Also, a higher value of this coefficient indicated more benefit. The interaction coefficient could be used as an indicator to quantify the benefit of ventilation reception and possibly be used as a criterion for comparison among various patient groups.

## Results

Data of 599,340 participants were analyzed, which encompassed 60,113 (10.0%) cases with ventilation therapy, 85,158 (14.2%) cases who died, and 514,182 (85.8%) cases who recovered. The mean (SD) age was 58.5 (18.3) [range = 18–114, being 58.3 (18.2) among women, and 58.6 (18.4) among men]. Characteristics of participants are presented in Table 1.

**Table 1:** Characteristics of participants.

Variable	N (%)
Demographics	
Sex	
Female	291,267 (48.6)
Male	308,073 (51.4)
Age	
18–39 years	113,211 (18.9)
40–64 years	240,298 (40.1)
More than 65 years	245,831 (41.0)
Underlying conditions	
CVD	
Yes	110,593 (18.5)
No	488,747 (81.5)
DM	
Yes	84,973 (14.2)
No	514,367 (85.8)
COPD	
Yes	26,153 (4.4)
No	573,187 (95.6)
Malignancy	
Yes	10,610 (2.0)
No	588,730 (98.0)
Outcomes	
Ventilation therapy	60,113 (10.0)
Death	85,158 (14.2)
Recovery	514,182 (85.8)

**Figure 1:**
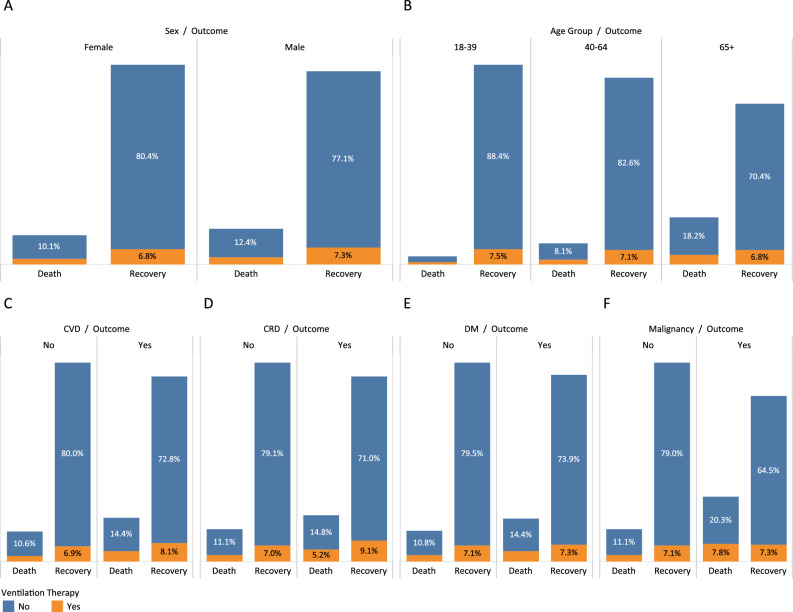
COVID-19 outcome based on (**A**) sex, (**B**) cardiovascular disease, (**C**) chronic respiratory disease, (**D**) malignancy, (**E**) diabetes mellitus, and (**F**) age groups.

The COVID-19 outcome based on sex, age-groups and underlying diseases are presented in Fig. 1.

Among all combinations, analysis of eight groups was not available due to paucity of data (Table 2).

**Table 2:** The estimated benefit of ventilation therapy among patients of different age groups with various underlying conditions.

id	COPD	Malignancy	CVD	DM	Age	Benefit^a^	*P*-value	Observation (Total^b^)	Observation (Ventilation^c^)	Observation (Recovery^d^)
1	No	No	No	No	18–39	1.983	< 0.001	103,991	8574	100,269
2	No	No	No	No	40–64	1.728	< 0.001	171,041	14,566	155,469
3	No	No	No	No	65 +	1.369	< 0.001	139,453	14,014	108,579
4	No	No	No	Yes	18–39	1.267	N/S	2827	288	2654
5	No	No	No	Yes	40–64	1.455	< 0.001	22,096	2214	19,465
6	No	No	No	Yes	65 +	1.72	< 0.001	23,258	2705	17,752
7	No	No	Yes	No	18–39	1.836	0.008	2895	336	2667
8	No	No	Yes	No	40–64	1.31	< 0.001	22,712	2539	20,251
9	No	No	Yes	No	65 +	1.266	< 0.001	43,934	5722	34,249
10	No	No	Yes	Yes	18–39	− 2.412	N/S	401	46	361
11	No	No	Yes	Yes	40–64	1.877	< 0.001	10,660	1276	9088
12	No	No	Yes	Yes	65 +	1.427	< 0.001	19,903	2598	15,205
13	No	Yes	No	No	18–39	3.639	< 0.001	1136	136	858
14	No	Yes	No	No	40–64	1.255	0.004	3788	528	2816
15	No	Yes	No	No	65 +	0.691	N/S	3085	501	2098
16	No	Yes	No	Yes	18–39	N/A	N/A	16	2	15
17	No	Yes	No	Yes	40–64	− 0.324	N/S	313	44	242
18	No	Yes	No	Yes	65 +	2.592	N/S	388	72	258
19	No	Yes	Yes	No	18–39	N/A	N/A	21	6	16
20	No	Yes	Yes	No	40–64	− 0.808	N/S	234	44	181
21	No	Yes	Yes	No	65 +	1.867	N/S	617	111	440
22	No	Yes	Yes	Yes	18–39	N/A	N/A	7	1	6
23	No	Yes	Yes	Yes	40–64	4.885	N/S	132	16	99
24	No	Yes	Yes	Yes	65 +	3.79	0.049	279	45	206
25	Yes	No	No	No	18–39	0.499	N/S	1685	238	1582
26	Yes	No	No	No	40–64	0.873	0.041	5958	721	5204
27	Yes	No	No	No	65 +	0.466	N/S	7095	1015	5391
28	Yes	No	No	Yes	18–39	241.684	N/S	44	8	40
29	Yes	No	No	Yes	40–64	3.137	0.005	834	123	696
30	Yes	No	No	Yes	65 +	0.605	N/S	1295	172	977
31	Yes	No	Yes	No	18–39	445.595	N/S	131	14	118
32	Yes	No	Yes	No	40–64	0.651	N/S	1599	221	1348
33	Yes	No	Yes	No	65 +	0.76	0.032	4488	730	3329
34	Yes	No	Yes	Yes	18–39	N/A	N/A	21	3	18
35	Yes	No	Yes	Yes	40–64	0.934	N/S	698	105	572
36	Yes	No	Yes	Yes	65 +	2.473	< 0.001	1711	277	1274
37	Yes	Yes	No	No	18–39	− 383.91	N/S	34	6	25
38	Yes	Yes	No	No	40–64	6.504	0.017	183	31	120
39	Yes	Yes	No	No	65 +	− 0.061	0.982	182	29	120
40	Yes	Yes	No	Yes	40–64	N/A	N/A	14	1	11
41	Yes	Yes	No	Yes	65 +	− 338.243	1.00	31	4	22
42	Yes	Yes	Yes	No	18–39	N/A	N/A	1	0	1
43	Yes	Yes	Yes	No	40–64	− 6.389	1.00	24	5	15
44	Yes	Yes	Yes	No	65 +	− 3.169	0.494	80	16	49
45	Yes	Yes	Yes	Yes	18–39	N/A	N/A	1	1	1
46	Yes	Yes	Yes	Yes	40–64	N/A	N/A	12	2	9
47	Yes	Yes	Yes	Yes	65 +	1466.119	0.999	32	7	16

^a^The interaction coefficient of the second model.

^b^The total number of patients with COVID-19 who were admitted to the hospital in each group.

^c^The total number of patients with COVID-19 who received ventilation therapy in each group.

^d^The total number of patients with COVID-19 who recovered after hospital admission in each group.

**Figure 2:**
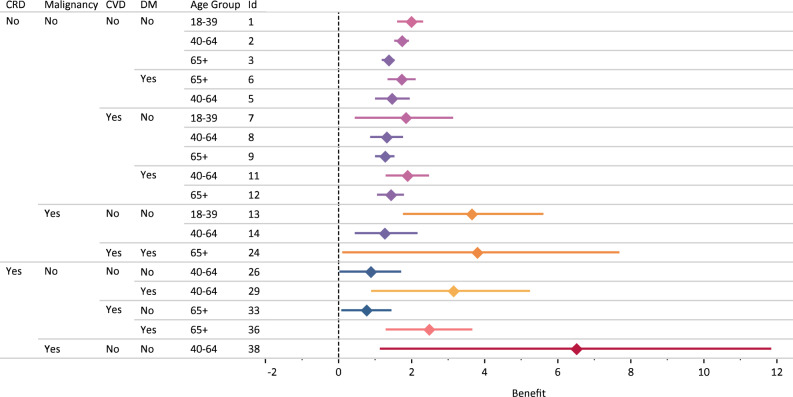
Patient groups who significantly benefited from ventilation therapy: estimated benefit (95% Confidence Interval).

Among all groups with sufficient data for analysis, patients aged 40–64 years who had CRD and malignancy benefitted the most from ventilation therapy; followed by patients aged 65 + years who had malignancy, CVD, and DM; and patients aged 18–39 years who had malignancy. Patients aged 65 + who had CRD and CVD gained the least benefit from ventilation therapy. Among patients with DM, patients aged 65 + years benefited from ventilation therapy, followed by 40–64 years. Among patients with CVD, patients aged 18–39 years benefited the most from ventilation therapy, followed by patients aged 40–64 years and 65 + years. Among patients with DM and CVD, patients aged 40–64 years benefited from ventilation therapy, followed by 65 + years. Among patients with no history of CRD, malignancy, CVD, or DM, patients aged 18–39 years benefited the most from ventilation therapy, followed by patients aged 40–64 years and 65 + years (Fig. 2).

## Discussion

This longitudinal study provides new insights on optimizing the strategies for ventilation therapy prioritization among patients with COVID-19, based on the real-world data of nearly 600,000 hospitalized patients with COVID-19. So far, there has been focus on how to prioritize patients with COVID-19 for ventilation therapy^19^. Nevertheless, there has not been much evidence on how much patients of different age groups with various underlying conditions actually benefitted from ventilation therapy based on real-world data. Some studies made endeavours to predict COVID-19 severity^20,21^ or the need for mechanical ventilation^21,22^; however, their approaches have not been investigated in the real-world to determine their outcomes.

In this study, patients aged 40–64 years who had CRD and malignancy benefitted the most from ventilation therapy, followed by patients aged 65 + years who had malignancy, CVD, and DM; and patients aged 18–39 years who had malignancy. Considering that these patient groups are considered to be at moderate or high risk of severe COVID-19 and possibly require ventilation therapy^23^, it was propitious that ventilation therapy could increase their chance of recovery.

Patients with COVID-19 who have DM are more likely to require mechanical ventilation^24^. Among patients with DM, older age is associated with worse COVID-19 outcomes^25,26^. In this study, patients with DM aged 65 + years benefited from ventilation therapy more than those aged 40–64. It is worth mentioning that all patients aged 40 + who only had DM benefitted from ventilation therapy.

Pre-existing CVD is independently associated with COVID-19 adverse outcomes^27^. Among patients who only had a CVD in this study, the younger the patients, the more they benefitted from ventilation therapy, unlike what was witnessed for DM. The same age pattern was also seen among patients who had DM and CVD. Some guidelines include age group as an additional consideration^19^.

Individuals also usually prioritize younger patients in situations of absolute scarcity of life sustaining resources; however, simply excluding patients from prioritization solely based on their age could be ethically unjustified and biased against older adults^28^. Although age-based discrimination includes moral conflicts and socio-cultural issues, ageism has become more apparent since the beginning of the COVID-19 pandemic. The media has played a significant role in this sense, while broadcasting discussions on the age limits for intensive care and ventilation allocation, unintentionally implying that an older person's life is worth less than a young person's^29^. In this study, among otherwise healthy patients, patients aged 18–39 years benefited the most from ventilation therapy, followed by patients aged 40–64 years, and patients aged 65 + years.

While COVID-19 has resulted in severe shortage of ventilators^6^ worldwide, countries with limited resources face the most challenges to serve all clinically eligible patients during the pandemic^8^. In this sense, factoring the level of benefit each patient would receive from ventilation therapy could help optimizing current guidelines.

In a study, the public opinion on priorities towards the fair allocation of ventilators during the COVID-19 pandemic was investigated, where people assigned a high priority score to patients with underlying diseases^30^. This could imply that people assumed that ventilation therapy would generally improve the outcome for patients with underlying conditions. Nevertheless, the real-world data suggested that patients' age group and underlying diseases could play a significant role in the outcome of ventilation therapy. This calls for knowledge translation by public health authorities and the media to regularly convey the prognostic factors of COVID-19 based on emerging evidence to justify people's expectations from the healthcare systems.

In a Delphi study, a panel of experts were asked to prioritise the allocation of ventilators based on various medical or non-medical factors. While the panel considered patients with active-malignancy to have low priority in receiving ventilation therapy, the real-world data made it crystal clear that patients with malignancy could also benefit from ventilation therapy. Moreover, the panel did not reach a consensus regarding underlying diseases^31^. The deviation of real-world data from the experts' perspectives highlights the potential bias the physicians could have when making a death-life decision, which needs to be taken into account by future guidelines on the fair allocation of ventilators.

Some guidelines assess patients based on their clinical condition at admission, which could include assessment of irreversible shock, and mortality risk using the Sequential Organ Failure Assessment (SOFA) score^32^. They also recommend continuous evaluation for withdrawing patients whose clinical condition is not improving despite ventilation therapy^7^. Nevertheless, few studies have assessed the application of the current triage criteria to actual patients. In the early days of the pandemic, a retrospective cohort study highlighted how divergent even supposedly similar triage approaches could be, suggesting that different triage approaches identified substantially other patients for initial consideration for withholding or early withdrawal of mechanical ventilation^33^. We did not find any studies that investigated the role of ventilation therapy in improving the course of COVID-19 in a setting where patients have been triaged based on SOFA scores.

### Strengths and limitations

This is the first nationwide study to quantify the benefit of ventilation therapy based on the real-world data around 600,000 hospitalized patients of various age groups with COVID-19 who had DM, CVD, malignancy, or CRD. The strength of this study lies in a large sample and data gathering since the early days of the outbreak in Iran. Findings could empower public health authorities to optimize the ventilation therapy prioritization strategies among patients with COVID-19 admitted to hospitals, especially considering that there are currently no national guidelines for allocation of ventilators at the time of resources scarcity in Iran and the decision to prioritize patients for ventilator allocation is performed based on hospital regulations. The data period for this study spans a relatively long period of time, covering multiple waves of the COVID-19 pandemic. We acknowledge the importance of analyzing the impact of varying infection situations on healthcare stress. In our methodology, we have taken this into account by incorporating environmental controls in our models. Specifically, we divided the hospitalization periods into several intervals to capture the fluctuations in healthcare stress and resource availability. These intervals were selected based on the occurrence of distinct waves and the associated demands on medical care. The analyzed intervals in our study include February–March 2020, April–May 2020, June-July 2020, August–September 2020, November–December 2020, January–February 2021, March–April 2021, and May–June 2021. By considering these intervals, we aimed to address the potential variations in stress on healthcare systems and the availability of resources across different phases of the pandemic. This approach allows us to account for the dynamic nature of the COVID-19 situation and its impact on our study outcomes.

We realize the limitations of the study. Due to the lack of a national integrated electronic health records system in Iran, many underlying conditions or baseline data of patients, such as their body mass index or behavioral risk factors, were not properly recorded in the COVID-19 registry. In this study, we divided the age groups into three categories: 18–39 years, 40–64 years, and 65 + years. The inclusion of the elderly population as a separate age group (65 +) is supported by its significant health and economic burden. This age group is known to have higher vulnerability and specific healthcare needs during the COVID-19 pandemic. Additionally, we chose to include a separate age group of 40–64 years due to the onset of chronic diseases typically occurring around this age range. This group is at a different stage of life compared to the elderly population, and the prevalence of chronic conditions is relatively higher. By considering this age group separately, we aimed to capture any potential differences in the impact of ventilation therapy among individuals with CRD and comorbidities. The age group of 18–39 years was included as another distinct category due to the relatively lower expectation of chronic diseases and comorbidities within this age range. This group serves as a reference for comparison and allows us to examine the potential benefits of ventilation therapy in a younger population without significant pre-existing health conditions. Despite the large study population, data points for some patient groups were insufficient for analysis, which need to be addressed in future studies. Moreover, the current study focused on investigating the clinical benefit of ventilation therapy among different patient groups with COVID-19 based on real-world data. Although we recognize the importance of incorporating health economic parameters, such as improvement in life expectancy or Quality Adjusted Life Years (QALYs), in policy discussions, we did not directly address these parameters in our analysis due to lack of forthcoming data. Future studies should aim to integrate health economic perspectives to evaluate the cost-effectiveness and long-term outcomes associated with different ventilator allocation strategies. This would provide policymakers with a more comprehensive understanding of the potential benefits and costs of alternative approaches.

### New insights and conclusion

The results of this study could have a significant message: should the prioritization guidelines for ventilators allocation take no notice of the real-world data, patients might be deprived of ventilation therapy, who could benefit the most from it. The comparison of real-world evidence with the general population's attitudes and medical experts showed an unexpected bias against older age groups and underlying conditions. This study promotes a new aspect of treating patients for ventilators as a scarce medical resource, considering whether ventilation therapy would improve the patient's clinical outcome. This gains significance considering the divergent outcomes of existing guidelines, especially for patients meeting the lowest priority criteria for mechanical ventilation^33^. As a rapidly evolving crisis, numerous therapeutic or preventive approaches are being investigated to lessen the burden of the COVID-19 pandemic^34,35^. It could be suggested that rather than focusing on the scarcity of ventilators, guidelines focus on evidence-based decision-making algorithms to also take the usefulness of the intervention into account, similar to some other medications, whose beneficial effect is dependent on the selection of the right time in the right patient^36^.

## Data Availability

De-identified, individual participant data will be made available upon requests directed to the corresponding author; after the approval of a proposal, data can be shared through a secure online platform.
